# Ammonium Removal from Aqueous Solutions by Clinoptilolite: Determination of Isotherm and Thermodynamic Parameters and Comparison of Kinetics by the Double Exponential Model and Conventional Kinetic Models

**DOI:** 10.3390/ijerph9030970

**Published:** 2012-03-19

**Authors:** İsmail Tosun

**Affiliations:** Department of Environmental Engineering, Süleyman Demirel University, Isparta 32260, Turkey; Email: ismailtosun@sdu.edu.tr; Tel.: +90-246-211-1855; Fax: +90-246-237-0859

**Keywords:** adsorption, ammonium, clinoptilolite, double exponential model, isotherm

## Abstract

The adsorption isotherm, the adsorption kinetics, and the thermodynamic parameters of ammonium removal from aqueous solution by using clinoptilolite in aqueous solution was investigated in this study. Experimental data obtained from batch equilibrium tests have been analyzed by four two-parameter (Freundlich, Langmuir, Tempkin and Dubinin-Radushkevich (D-R)) and four three-parameter (Redlich-Peterson (R-P), Sips, Toth and Khan) isotherm models. D-R and R-P isotherms were the models that best fitted to experimental data over the other two- and three-parameter models applied. The adsorption energy (E) from the D-R isotherm was found to be approximately 7 kJ/mol for the ammonium-clinoptilolite system, thereby indicating that ammonium is adsorbed on clinoptilolite by physisorption. Kinetic parameters were determined by analyzing the *nth*-order kinetic model, the modified second-order model and the double exponential model, and each model resulted in a coefficient of determination (R^2^) of above 0.989 with an average relative error lower than 5%. A Double Exponential Model (DEM) showed that the adsorption process develops in two stages as rapid and slow phase. Changes in standard free energy (∆G°), enthalpy (∆H°) and entropy (∆S°) of ammonium-clinoptilolite system were estimated by using the thermodynamic equilibrium coefficients.

## 1. Introduction

Discharging of wastewater streams containing high ammonium concentrations into the receiving body causes serious problems in the natural nutrient cycle between the living world and the soil, water, and atmosphere [1]. Hence, mitigation of contamination caused by ammonium compounds from wastewater has a vital importance with regard to fresh water usage [2]. Although a number of processes such as air stripping, breakpoint chlorination, nitrification-denitrification, and ion exchange have been proposed for the removal of ammonia from the environment and industrial water systems, only few methods such as biological and physicochemical treatment are commonly applicable for the direct control of ammonia in wastewaters [2,3].

In the last three decades, an increasing number of investigations have been conducted on ammonium removal from wastewater by ion exchange because of the ammonium selectivity of clinoptilolite [4,5,6,7]. Demir *et al*. [8] examined the ammonium removal characteristics of natural zeolite by using a packed bed. Sarıoglu [9] considered the removal of ammonium from municipal wastewater using natural Turkish (Dogantepe) zeolite. Ramos *et al*. [10] studied the effects of temperature and solution pH on ammonium ion exchange capacity of clinoptilolite obtained from mineral deposits located in San Luis Potosi and Sonora, Mexico. Besides these studies carried out to remove ammonium from the existing environment, zeolites have also been examined in a variety of applications such as reducing the potential health risks of carcinogens caused by smoking [11], increasing the compost quality by trapping ammonium and reducing nitrogen losses from the compost [12].

Clinoptilolite is an abundant natural zeolite found in igneous, sedimentary and metamorphic deposits in the form of alumino-silicate minerals with high cation-exchange capacity. The adsorption capacity of clinoptilolite is significantly affected by physical and chemical pretreatment and loading or regeneration of clinoptilolite. The pretreatment of natural zeolites by acids, bases, surfactants, *etc*., is an important method to improve their ion-exchange capacity [8,13,14,15,16]. Practically, the result of any pretreatment operation is the increase of the content in a single cation, what is called homoionic form. Therefore, prior to any ion-exchange application, certain ions from the structure of the material are removed by pretreatment and more easily removable ones are located [17,18].

Isotherms and kinetic results are valuable information to determine the suitability and effectiveness of the adsorption process [19]. Aksu and İşoğlu [20] examined the mechanisms of biosorption and potential rate-controlling steps including external mass transfer, intraparticle diffusion and biosorption process. They found that for all initial copper(II) concentrations initial sorption of copper(II) occurred rapidly and the majority of copper(II) uptake occurred within the first 30 min. Betul *et al*. [21] stated that microbial metal uptake generally involves the rapid uptake stage followed by a slow uptake.

Although many studies have been conducted on adsorption isotherm and kinetics, very few works [22,23,24] in the literature have been presented on the evaluation of adsorption stages. Actually, adsorption takes places rapidly on available interior surfaces of the pores in clinoptilolite that are easily accessible at the initial stages of the adsorption process. Then, the adsorption process continues slowly due to limitation in available surface sizes. This two-step adsorption mechanism, the rapidly and slowly absorbed fractions, can be described by a Double Exponential Model (DEM) that can also be used in water and wastewater treatment process optimizations.

The main objective of the present study is to examine ammonium removal by clinoptilolite, which was initially pretreated with aqueous sodium chloride solution, and to analyze the equilibrium modeling by two- and three-parameter adsorption isotherms, the kinetic modeling by *nth*-order reaction, modified second-order and double exponential model, and the thermodynamic parameters of the ammonium removal.

## 2. Material and Methods

### 2.1. Physical Properties of the Clinoptilolite

Clinoptilolite used in the experiments was obtained from Balıkesir in the Northwestern part of Turkey. The chemical properties of the clinoptilolite can be found in our previous study [14]. Clinoptilolite was ground down to a grain size range of 0.30–0.60 mm. Prior to the batch adsorption experiments, clinoptilolite was washed with distilled water to remove surface dust and then dried in an oven at 70 °C. Subsequently, it was treated with 2 M NaCl solution at 22 °C by shaking for a period of 24 h to activate its pores and dried again.

### 2.2. Experimental Procedure

Synthetic samples were prepared to give NH_3_-N concentrations of 30, 60, 100, 160 and 250 mg/L by adding required NH_4_Cl salt to distilled water for both isotherm and kinetic studies. For kinetic studies, samples of 5 g were equilibrated with 500 mL ammonium nitrogen solution at 10, 25, and 40 °C for 100 min. Samples were periodically taken for measurement of aqueous phase of ammonia concentrations. Batch mode adsorption isotherm studies were carried out in conical flasks containing 200 mL of the solutions and 2 g clinoptilolite at temperatures of 10, 25, and 40 °C. The flasks were placed in a magnetic stirrer and agitated for 4 h at a fixed agitation speed of 200 rpm. All experiments were carried out at an initial pH of 4.5 where clinoptilolite ion exchange occurs conveniently when the pH is between 4 and 8 in which range ammonium is at ionized form [3]. The final pH value was also monitored and it was found to be below 7.5 for all experiments. Ammonium nitrogen that remained in the solution of the sample was determined using the classic Nessler procedure [25].

The amount of ammonium nitrogen in the solid phase, Q (mg/g), was calculated by using the following equation:


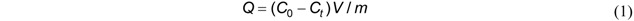


where C_0_ and C_t_ are the initial and retained ammonium concentration (mg/L) in solution at time t, respectively; *V* is the solution volume (mL); and *m* is the weight of adsorbent (g).

### 2.3. Statistical Analysis of Data

The adsorption isotherm and adsorption kinetic parameters were determined by using non-linear regression analysis. The non-linear method is a better way to obtain the kinetic parameters than the linear method, and thus it should be primarily adopted to determine the kinetic parameters [26]. A minimization procedure using the solver add-in function of the Microsoft Excel has been adopted to solve isotherm and kinetic equations by minimizing the hybrid fractional error function (HYBRID) between the predicted values and the experimental data [27].


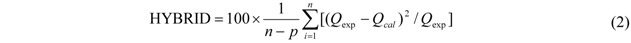


where the subscripts “exp” and “cal” denote the experimental and calculated values of *Q*, respectively; *n* is the number of data points; and *p* is the number of parameters in kinetic equation.

In order to quantitatively compare the applicability of isotherm and kinetic models for fitting the experimental data, non-linear coefficient of determination (R^2^) and average relative error (*∆Q*) [28] were calculated:


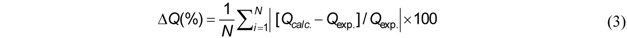


**Table 1:** Expression of two-parameter and three-parameter adsorption isotherm models.

Isotherm models	Expression *	Adjustable model parameters *	Constraints *
**Two-parameter isotherms**			
Freundlich [29]	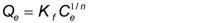	*K_f_, 1/n*	*n* > 1
Langmuir [30]	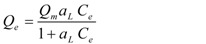	*Q_m_, a_L_*	-
D-R [31,32]	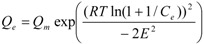	*Q_m_, E*	*Q_m_*, *E* > 0
Tempkin [33]	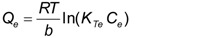	*K_Te_, b*	-
**Three-parameter isotherms**			
Redlich-Peterson [34]	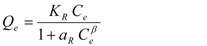	*K_R_, a_R_, β*	0 < *β* < 1
Sips [35]	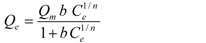	*Q_m_, 1/n, b*	0 < *1/n* < 1
Toth [36]	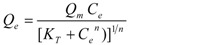	*Q_m_, K_T_, 1/n*	0 < *1/n* < 1
Khan [37]	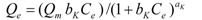	*Q_m_, b_K_, a_K_*	

* where; Q_e_ is equilibrium solid phase concentration (mg/g) and C_e_ is equilibrium liquid phase concentration (mg/L) in all isotherm models. In all models, *Q_m_* parameter is relevant with adsorption capacity. In Freundlich isotherm model *K_f_* and *n* are isotherm parameters characterizing adsorption capacity and intensity, respectively. In Langmuir equation *K_L_* and *a_L_* are the Langmuir constants related to the adsorption capacity and energy of adsorption, respectively. In D-R isotherm, E is energy of adsorption. In Tempkin isotherm *K_Te_* is equilibrium binding constant (L/g), *b* is related to heat of adsorption (J/mol), *R* is the gas constant (8.314 × 10^−3^ kJ/K mol) and *T* is the absolute temperature (K). *K_R_* (L/g) and *a_R_* (L/mg) are Redlich–Peterson isotherm constants and *β* is the exponent which lies between 0 and 1. In Sips isotherm, *a_S_* constant related to energy of adsorption and *1/n* is exponent. *K_To_* is the Toth model constant and n the Toth model exponent (0 < *n* ≤ 1). *b_K_* is the Khan model constants and *a_K_* is the Khan model exponent.

## 3. Results and Discussion

### 3.1. Adsorption Isotherms

Adsorption isotherms describe the relationship between the amount of adsorbed ion on adsorbent and the final ion concentration in the solution. Experimental results have been analyzed using four two-parameter isotherm models including Freundlich, Langmuir, Tempkin and D-R; and four three-parameter adsorption isotherm models including R-P, Sips, Toth and Khan isotherm models (Table 1) [29,30,31,32,33,34,35,36,37].

**Figure 1:**
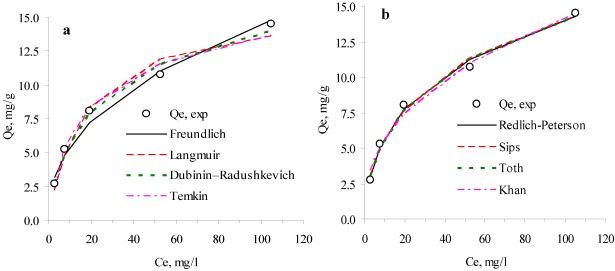
Comparison of adsorption isotherm models at 10 °C; (**a**) two-parameter isotherms; (**b**) three-parameter isotherms.

Figure 1 illustrates the two- and three-parameter isotherm models that are fitted to the experimental data obtained at 10 °C. Similar trends were also obtained at 25 °C and 40 °C (results not shown). Increase in temperature didn’t cause any significant changes in the adsorption capacity of clinoptilolite which was found to be 14.50, 14.50, and 13.88 mg/g for 10, 25, and 40 °C, respectively.

The determined isotherm parameters obtained for all temperatures are shown in Table 2. The isotherm constants, HYBRID, average relative errors (ΔQ_e_, %), and coefficient of determination (R^2^) based on the actual deviation between the experimental points and predicted values are also given. As given in Table 2, even though all the R^2^ values are above 0.96 which indicates best fit the R^2^ values for two-parameter isotherms are slightly smaller than that of three-parameter isotherms. However, when the average relative error of three-parameter isotherms are inspected it can be seen that they are clearly lower than that of two-parameter isotherms. This shows average relative error criteria is more pronounced compared to R^2^ criteria to evaluate the experimental results. Gunay [38] also stated that three-parameter isotherm models resulted better performance than two-parameter models. On the basis of the ΔQ_e_ values obtained, out of all the two-parameter and three-parameter isotherm models, D-R isotherm and R-P isotherm, respectively, describe the experimental data effectively for all temparatures except at 40 °C ΔQ_e_ is slightly larger than that of Tempkin and Langmuir models. Calculated isotherm parameters in Langmuir, D-R, Sips and Toth equations are consistent with the experimental *Q_e_* values. When R^2^ values are considered, it was seen that the D-R isotherm and R-P isotherm models were also well fitted to the experimental data.

**Table 2:** Comparison of two-parameter and three-parameter isotherms for different temperatures.

Two-parameter isotherms	10 °C	25 °C	40 °C		Three-parameter isotherms	10 °C	25 °C	40 °C
**Freundlich**					**Redlich-Peterson**			
*K_f_*	2.055	1.961	1.660		*K*_R_	2.387	2.217	0.863
*1/n*	0.424	0.434	0.464		*a*_R_	0.610	0.585	0.084
R² (non-linear)	0.986	0.987	0.962		*β*	0.709	0.701	0.905
HYBRID	7.211	6.575	17.166		R² (non-linear)	0.993	0.994	0.999
ΔQ, (%)	8.093	7.692	12.487		HYBRID	27.832	25.424	14.187
**Langmuir**					ΔQ, (%)	4.799	4.458	4.048
*a*_L_	0.056	0.052	0.045		**Sips**			
*Q*_m_	15.902	16.163	16.305		*Q*_m_	33.075	33.905	17.338
R² (non-linear)	0.971	0.973	0.995		*a*_s_	0.058	0.054	0.051
HYBRID	92.123	85.887	14.316		*1/n*	0.551	0.560	0.913
ΔQ, (%)	9.868	9.620	3.438		R² (non-linear)	0.991	0.992	0.995
**D–R**					HYBRID	39.054	35.520	18.599
*Q*_m_	19.108	19.330	19.406		ΔQ, (%)	6.348	5.943	4.366
*E*	7.024	7.268	7.379		**Toth**			
R² (non-linear)	0.988	0.989	0.993		*Q*_m_	64.023	67.410	18.309
HYBRID	33.750	31.798	18.461		*K*_To_	1.540	1.631	11.042
ΔQ, (%)	3.980	3.504	5.888		*n*	0.256	0.258	0.798
**Tempkin**					R² (non-linear)	0.992	0.993	0.996
*K*_Te_	0.736	0.673	0.469		HYBRID	34.297	31.120	16.590
*b*	748.321	773.183	748.566		ΔQ, (%)	5.690	5.288	4.256
R² (non-linear)	0.982	0.981	0.999		**Khan isotherm**			
HYBRID	51.874	54.940	3.193		*Q*_m_	0.180	0.161	0.145
ΔQ, (%)	6.777	7.499	1.976		*b*_K_	545.242	548.264	545.194
					*a*_K_	0.599	0.589	0.583
					R² (non-linear)	0.988	0.989	0.969
					HYBRID	52.619	48.473	131.342
					ΔQ, (%)	8.141	7.727	13.157

The magnitude of energy of adsorption (E) in the D-R isotherm is around 1–16 kJ/mol and useful for the assessment of the adsorption mechanism. If this value is below 8 kJ/mol, physisorption is considered to occur. In the present case, the values of E slightly increased as the temperature increased from 10 to 40 °C and were found to be between 7.024 and 7.379 kJ/mol, which indicated physical adsorption.

### 3.2. Adsorption Kinetics

**Figure 2:**
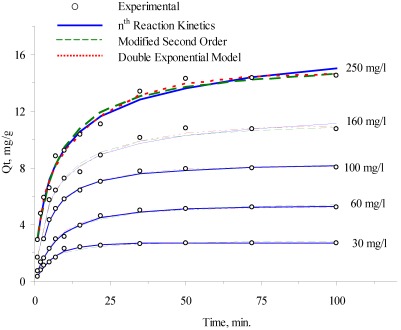
Comparison of adsorption kinetic models at 10 °C.

Adsorption kinetics are required for selecting optimum operational conditions of water and wastewater treatment facilities for full-scale processes. The results obtained from the experiments at 10 °C were examined to describe the reaction kinetics according to the *nth*-order kinetics, and the modified second-order and double exponential models. Figure 2 illustrates the kinetic models fitted to experimental data obtained at 10 °C. The determined kinetic parameters are shown in Table 3.

**Table 3:** Comparison of adsorption kinetic models at 10 °C.

C_i_ (initial conc.), mg/L	30	60	100	160	250
Q_e_, _exp._ (mg/g)	2.716	5.248	8.054	10.734	14.504
***nth*-order kinetic model**					
*Q_e_*, _cal._, (mg/g)	2.713	5.383	8.485	13.775	20.832
*n*	1.162	1.446	1.961	3.419	4.162
*βn*	0.996	1.014	0.857	0.848	1.085
*k_n_* (min^−1^)	0.173	0.123	0.231	0.220	0.174
HIBRID	0.654	0.532	0.205	1.474	2.918
Average Δq (%)	3.979	2.959	1.166	2.321	4.043
R^2^	0.989	0.996	0.997	0.989	0.987
**Modified second-order model**					
*Q_e_*, (mg/g)	2.961	5.819	8.518	11.499	15.621
*k_2_* (min^−1^)	0.228	0.138	0.236	0.175	0.141
*β*	0.901	1.002	0.839	1.004	1.113
HIBRID	0.892	0.607	0.176	1.624	3.154
Average Δq (%)	4.469	3.329	1.144	3.086	5.023
R^2^	0.980	0.993	0.997	0.988	0.986
**Double Exponential Model**					
*Q_e_*, (mg/g)	2.771	5.236	8.095	10.992	14.700
*D_1_*	26.427	33.567	45.292	50.886	53.777
*D_2_*	1.280	18.790	35.663	59.037	93.223
*K_D1_*	0.159	0.182	0.267	0.357	0.611
*K_D2_*	0.009	0.050	0.059	0.048	0.051
RF, %	95.38	64.11	55.95	46.29	36.58
SF, %	4.62	35.89	44.05	53.71	63.42
HIBRID	0.780	1.208	0.216	1.142	2.554
Average Δq (%)	4.682	3.903	1.233	2.516	2.841
R^2^	0.990	0.992	0.998	0.994	0.991

#### 3.2.1. *nth*-Order Kinetics

It is thought that instead of assuming order of the reaction as 1 or 2, the direct calculation of rate constant and order of the adsorption reaction is a more appropriate method [39]. Thus, *nth*-order kinetic model can be used. The model is expressed as follows [40]:


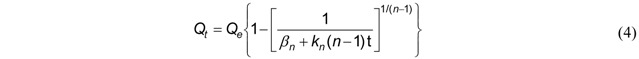


where *Q_e_* is the amount of ammonium adsorbed on the surface of the adsorbent at equilibrium, (mg/g); *Q_t_* is the amount of ammonium at any contact time, (mg/g); *k_n_* is the rate constant and its unit depends on the order of the reaction, (1/min) (mg/g)^1–n^, *β_n_* is related to impurities pre-adsorbed on the surface (*β_n_* = 1/(1 − *θ_0_*)^n−1^), *θ_0_* is surface coverage at pre-adsorbed stage (*θ_0_* = *Q_0_/Q_e_*), dimensionless.

The determined model parameters are listed in Table 3. The reaction order, *n*, increased with the increase in initial ammonium concentration, it was found to be between 1.162 and 4.162. This increase can be explained on the basis of the increase in driving force with the concentration gradient for the initially high ammonia concentration. In another study carried out by Özer [39], a similar finding to that obtained in the present study showed that the value of *n* in *nth*-order kinetics slightly decreased with an increase in initial temperature. Also, it is stated that the dominant mechanism depends on a combination of adsorbate and adsorbent and adsorption conditions such as temperature and concentration range [41]. The *k_n_* values were found to be in the range of 0.123 and 0.231 (1/min) (mg/g)^1–n^. *β_n_* values were approximately 1, which implies that there are no impurities or pre-adsorbed ammonium initially. 

#### 3.2.2. Modified Second-Order

Modified second-order equation can be obtained from the *nth*-order kinetics for n = 2 as follows [40]:


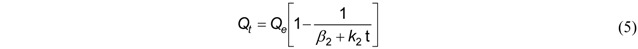


The modified second-order reaction constants *k_2_* and coefficient of determination R^2^ are presented in Table 3 for all initial ammonium concentrations. The values of *k_2_* were found to be in the range of 0.138–0.236 g/mg-min. *β_2_* values were about 1, as calculated in the *nth*-order kinetic equation.

#### 3.2.3. Double Exponential Model

The model describes the adsorption process with respect to both chemical and mathematical points of view [42], correlating the two-step mechanism as rapidly and slowly adsorbed fractions [43]. The model is expressed by the following equation:


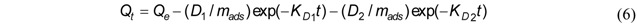


where D_1_ and D_2_ are the amount of rapidly and slowly adsorbed fraction of ammonium (mg/l), respectively, and K_D1_ and K_D2_ are rapid and slow rate constants (min^–1^). It should be noted that the sum of D_1_/m_ads_ and D_2_/m_ads_ has the same physical meaning as the calculated value of Q_e_, and K_D1_ is greater than K_D2_. Rapidly and slowly adsorbed fractions (%), RF and SF, can be calculated as: 


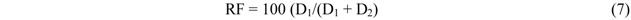


and


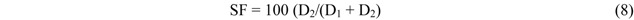


respectively.

Model parameters for the slow and rapid steps and statistical comparison parameters obtained from experimental data are presented in Table 3 for all initial concentrations. While RF values decreased with an increase in initial concentration, SF values increased with an increase in initial concentration. The reason for the decrease in the RF values is that adsorption takes place in restricted zones due to limitation in available surface sites of clinoptilolite for the high initial concentration. K_D1_ values increase with the initial concentration; meanwhile, K_D2_ values are almost the same. This finding is also in accordance with the observations of other similar studies [44,45]. Blázquez *et al*. [44] examined the kinetics of lead(II) biosorption by olive tree pruning waste found out that biosorption of lead(II) ions onto the biomass initially occurred within a fast removal rate stage, followed by a second slower removal rate stage, until reaching equilibrium. Ghaedi *et al*. [45] determined that the biosorption of Pb^2+^ ions by *Saccharomyces cerevisiae* took place in two stages: in the first stage, biosorption process in which 60–70% of the total process completed was achieved in 3 days, and in the second stage, equilibrium attained in 4 days. It was also suggested that in the first stage which was faster than the second stage, the Pb^2+^ ion was accumulated in the large available surface of biosorbent. The biosorption process was slowed down with the gradual occupation of surface binding sites.

#### 3.2.4. Evaluation of Kinetic Studies

For all kinetic models, calculated *Q_e_* values were almost the same as the experimental *Q_e_* values. Initially, the adsorption rate of ammonium by clinoptilolite was high up to 20–50 minutes, and then it gradually decreased with an increase in the contact time. According to ΔQ_e_ values, results of the kinetic studies show that the best fitted kinetic models are found to be in the following order: *nth*-order, double exponential and modified second-order kinetic models. Average relative errors are lower than 5%, and coefficients of determination are almost the same, thus indicating that these models effectively describe the adsorption process.

### 3.3. Thermodynamic Parameters

Thermodynamic parameters were evaluated by considering the thermodynamic equilibrium constants. The standard free energy were calculated using the following equation:


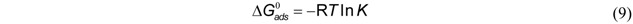


where *∆G°_ads_* is the free energy change (kJ/mol), *T* is the absolute temperature (K), R is the universal gas constant (8.314 × 10^−3^ kJ/K mol), *K* is the Langmuir constant, Tempkin constant or the thermodynamic equilibrium constants obtained using the method of Khan and Singh [46].

For the Tempkin and Langmuir isotherms, the values of Tempkin constant (*K_T_*) and Langmuir constant (*a_L_*), as given in Table 2 were used. In the Khan and Singh [46] method, the thermodynamic equilibrium constant (*K_KS_*) was calculated as follows:


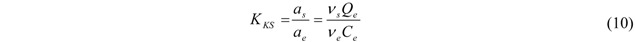


where *a_s_* is the activity of ammonium in solid phase, *a_e_* is the activity of ammonium in solution at equilibrium, *ν_s_* is the activity coefficient of the adsorbed ammonium and *ν_e_* is the activity coefficient of the ammonium in solution at equilibrium. As the ammonium concentration in the solution decreases and approaches zero, the activity coefficient ν approaches to unity. Equation (10) may be written as follows:


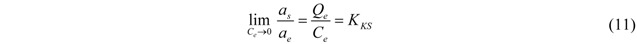


**Figure 3:**
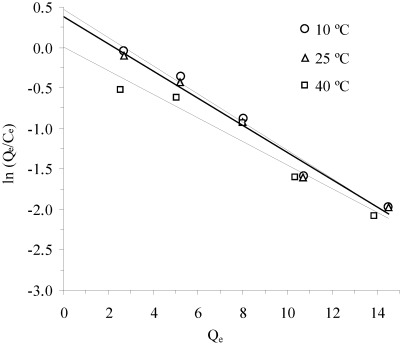
Plots of ln (Q*_e_*/*C_e_*) *vs*. *Q_e_* at various temperatures for the determination of thermodynamic equilibrium constants by using the method of Khan and Singh [46].

*K_KS_* can be obtained by plotting a straight line of ln(Q*_e_*/*C_e_*) *vs*. *Q_e_* (Figure 3) and extrapolating *Q_e_* to zero. Its intercept gives the values of *K_KS_*.

The other thermodynamic parameters, *i.e.*, the change in enthalpy (∆H°) and entropy (∆S°), were estimated from the following equation:


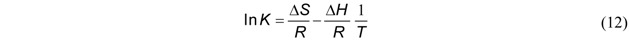


**Figure 4:**
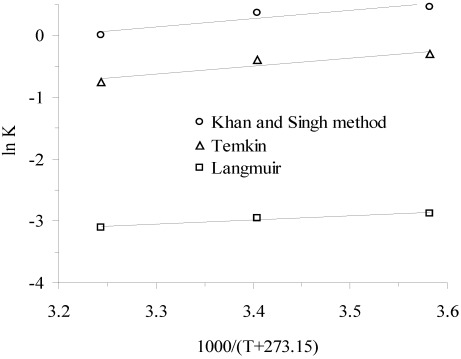
Plot of ln *K* *vs*. (1/T) for estimation of thermodynamic parameters.

The values of change in enthalpy (∆H°) and entropy (∆S°) were calculated from the slope and intercept of the plot of ln*K* *vs.* (1/*T*) (Figure 4).

The results of change in standard free energy, enthalpy and entropy are given in Table 4. The standard free energy of ammonium adsorption on clinoptilolite was found to be in the range of −1.10 to −0.03, 0.72 to 1.97 and 6.77 to 8.07 kJ/mol for Khan and Singh [46], Tempkin and Langmuir isotherms, respectively.

The positive values of ∆G° imply that the adsorption of ammonium on clinoptilolite is not spontaneous. ∆G° value increased with the temperature, indicating that the spontaneous nature of adsorption is inversely proportional to the temperature. Since the value of standard enthalpy change, ∆H°, is negative, the process is exothermic; therefore, an increase in the temperature leads to a lower adsorption of ammonium at equilibrium, and physical by nature and involves weak forces of attraction. The negative values of ∆S° suggest that the system exhibits random behavior.

**Table 4:** Comparison of thermodynamic parameters for adsorption of ammonia on Clinoptilolite.

Temperature, °C	*K* *	Slope × 10^−3^	Intercept	R^2^ (linear)	*∆G°*, kJ/mol	*∆H°*, kJ/mol	*∆S°*, J/mol-K
	***K_KS_***						
10 °C	1.596				−1.10		
25 °C	1.454	1.34	−4.20	0.877	−0.93	−11.11	−34.95
40 °C	1.011				−0.03		
	***K_Te_***						
10 °C	0.736				0.72		
25 °C	0.673	1.31	−4.90	0.873	0.98	−10.92	−40.73
40 °C	0.469				1.97		
	***a_L_***						
10 °C	0.056				6.77		
25 °C	0.052	0.65	−5.18	0.977	7.35	−5.43	−43.03
40 °C	0.045				8.07		

* Thermodynamic equilibrium constant.

## 4. Conclusions

The adsorption of ammonium on clinoptilolite was evaluated as a function of two- and three-parameter isotherms, adsorption kinetics and thermodynamic aspect. Generally, equilibrium data fitted better in three-parameter isotherm models than two-parameter isotherms models. D-R and R-P isotherms effectively described the experimental data for two-parameter and three-parameter isotherm models, respectively. Adsorption energy for ammonium–clinoptilolite system was found to be approximately 7 kJ/mol, which lies within the range of 1–8 kJ/mol for the physisorption processes, indicating that ammonium is adsorbed on clinoptilolite predominantly by physisorption process.

The kinetics of adsorption were found to conform to all kinetics studied with a good correlation. The best model that described the kinetic data was the *nth-*order kinetic model. Reaction order in the *nth*-order model increased with the initial concentration and was between 1 and 4. In the double exponential model, rapidly adsorbed fraction values decreased with an increase in initial concentration, and vice versa for slowly adsorbed fraction values. Thermodynamic parameters show that adsorption process was non-spontaneous and adsorption rate decreased with an increase in the temperature, thus showing the exothermic nature of the adsorption.
